# The Myths, Perils, and Pitfalls of Redo Pediatric Cardiac Surgery: The New Normal in Developing Countries Such as India

**DOI:** 10.7759/cureus.52642

**Published:** 2024-01-20

**Authors:** Vishal V Bhende, Tanishq S Sharma, Mathangi Krishnakumar, Anikode Subramanian Ramaswamy, Kanchan Bilgi, Sohilkhan R Pathan

**Affiliations:** 1 Pediatric Cardiac Surgery, Bhanubhai and Madhuben Patel Cardiac Centre, Shree Krishna Hospital, Bhaikaka University, Karamsad, IND; 2 Community Medicine, SAL Institute of Medical Sciences, Ahmedabad, IND; 3 Anaesthesiology, St. John's Medical College Hospital, Bengaluru, IND; 4 Pathology, PES Institute of Medical Sciences and Research, Kuppam, IND; 5 Neuroanaesthesiology, People Tree Hospitals, Bengaluru, IND; 6 Clinical Research, Bhanubhai and Madhuben Patel Cardiac Centre, Shree Krishna Hospital, Bhaikaka University, Karamsad, IND

**Keywords:** echocardiography, birth defects, epidemiology, congenital heart defects, redo-surgery

## Abstract

Pediatric patients undergoing reoperative cardiac surgery after a previous sternotomy face a higher degree of surgical complexity compared to those undergoing initial procedures. They have higher intraoperative and postoperative risks. The increased risk of surgery is due to preoperative patient factors and intraoperative technical challenges. Redo-pediatric cardiac surgery is a common event in almost every pediatric cardiac surgeon's professional life. Redo-surgery is almost inevitable in patients who have multi-stage repair of congenital heart surgeries and biological valves at a young age, and often in those having valve repair in rheumatic disease. So, being familiar with the pitfalls and precautions to be taken is of crucial importance. In general, the patients presenting for repeat procedures are sicker, older, and have more comorbid conditions. The dissection is always rendered difficult by adhesions, scarring, and previous graft placements. Hence, prolonged dissection time, intraoperative injuries to heart chambers, great vessels, and grafts, increased bleeding, and poorer cardiac function result in higher morbidity and mortality in such subsets of patients. The outcome is worse with emergency redo-cardiac surgeries.

## Editorial

The economic implications of repeat surgeries for congenital heart disease at the microlevel remain largely unexplored, especially in resource-constrained settings, a common scenario in developing nations such as India. Congenital heart disease (CHD) constitutes approximately 33% of major birth anomalies; approximately 9.3 in 1000 newborns are affected [1]. Children diagnosed with CHD often necessitate surgical and ongoing medical care. Despite notable medical and surgical advancements in the treatment of CHD, the associated expenses have escalated. Hospital expenditures linked to congenital cardiovascular anomalies reportedly surpass half of all costs related to birth defects [2]. The benchmark for cost-effective intervention in the United States is set at $50,000 for one year of quality-adjusted life, while in England, it adheres to the National Institute for Health and Clinical Excellence (NICE) guidelines, which recommend around £20,000. These countries have economies that are significantly larger than ours, with gross domestic products (GDPs) exceeding ours by a factor of ten or more. Similarly, their healthcare spending dwarfs ours, being over 100 times greater. Consequently, treating CHD is theoretically considered a highly effective intervention based on public health criteria. However, despite its conceptual effectiveness, over 50% of families may still find it financially challenging to cover the associated costs. Even if affordability is not an issue, our current manpower resources are insufficient to meet more than 10% of the existing demands [3]. In the latest Union Budget for 2023, the health sector was allocated 88,956 crore rupees, equivalent to 889.56 billion US dollars (current exchange rate of 1 USD = 81.89 Indian rupee (INR)). How effectively this can be used for CHD patients remains a question, as in our country, the priority is still the many communicable and noncommunicable diseases. The disease burden of CHD is largely unrecognized. Table 1 depicts the list of redo-pediatric cardiac surgery procedures.

**Table 1:** The list of redo surgeries in pediatric cardiac surgery SV: Single ventricle, RV: Right ventricle, PA: Pulmonary artery, TOF: Tetralogy of Fallot, RVOTO: Right ventricular outflow tract obstruction, LVOTO: Left ventricular outflow tract obstruction, VSD: Ventricular septal defect, RVOT: Right ventricular outflow tract, DORV: Double outlet right ventricle, ALCAPA: Anomalous left coronary artery from the pulmonary artery, TAPVC: Total anomalous pulmonary venous connection

No.	Description
1.	Staged procedures: Single-ventricle (SV)/univentricular physiology palliation pathway
2.	Conduit change procedures: Change of right ventricle (RV); pulmonary artery (PA) conduit done previously
3.	Baffle obstruction in modified Senning's procedure
4.	Failed procedures
	(a) Failure of valve repair viz. repair of complete atrioventricular canal repair as a primary surgery
	(b) Mitral valve repair or aortic valve repair in rheumatic heart disease in the first-stage
5.	Neopulmonary artery reconstruction stenosis at bifurcation level or branch PAs level in case of previous arterial switch operations
6.	Surgical implantation of pulmonary valve for patients having severe pulmonary insufficiency after tetralogy of Fallot (TOF) repair
7.	Redo-relief of right ventricular outflow tract obstruction (RVOTO) in procedures other than TOF
8.	Relief of left ventricular outflow tract obstruction (LVOTO) after primary surgery viz. previous atrioventricular canal repair
9.	Ventricular septal defect (VSD) closure related: Recurrent or residual RVOT narrowing after VSD closure surgery, tricuspid regurgitation following VSD closure, residual or additional VSD missed at primary surgery
10.	Baffle obstruction after tunnel repair for double outlet right ventricle (DORV)
11.	Coronary issues following previous arterial switch operation, anomalous left coronary artery from the pulmonary artery (ALCAPA) repair
12.	Redo following total anomalous pulmonary venous connection (TAPVC) repair, residual or recurrent pulmonary venous narrowing
13.	Residual subaortic membrane requiring redo-surgery
14.	Redo for infective endocarditis of native or previous patch or native or previous valve
15.	Pacemaker battery/generator changes in patients with complete heart block
16.	Patients waiting for heart transplant programs, or patients who have undergone primary surgery for SV physiology and hit a roadblock as alternative surgical options are fraught with additional risks due to unfavorable anatomy

As per Table 1, staged procedures for univentricular physiology for pulmonary stenosis include a modified Blalock-Taussig (BT) shunt if branch pulmonary arteries (PAs) are small or a bi-directional Glenn (BDG) shunt if branch PAs are fair to good-sized. Patients presenting with pulmonary arterial hypertension on the single-ventricle (SV) pathway have to undergo PA banding followed by BDG. The final stage of the SV pathway is the completion of the Fontan, which requires a Gore-Tex conduit (WL Gore & Associates Inc., Newark, DE, USA) for bridging the inferior vena cava (IVC) to the PAs.

Through this staging procedure, the patient is relieved of cyanosis until the last stage. The journey of patients from the first stage to the last stage of surgery might stretch over a lengthy period of 10 to 15 years in the life span of the individual. The procedures undertaken for complete atrioventricular canal defect (AVCD) valve repairs in infants and the rheumatic population indicate that these patients will require a redo-surgery for valvular repairs. In AVCD repairs, the mitral regurgitation (MR) severity often leads to redo surgery. The other subset of patients who have conduit requirements faces difficulty in first procuring the ideal conduit and then facing challenges with conduit replacements. In the lifespan of one individual, three to four conduit replacements are mandatory, leading to morbidity and, sometimes, mortality. The cost of conduits also differs across the spectrum. Commercial conduits are offered in sizes ranging from 12 to 18 mm, with an approximate cost of 1.5 to 2 lakh rupees plus 12% goods and services tax (GST), which totals up to 1.68 to 2.12 lakh rupees (2018.77 US dollars).

These expenses are borne by hospitals as they are not covered by existing government scheme packages. In the rare instances where alternative conduits, such as dacron conduits, are utilized, they are typically 14 mm in size. A 30-cm-long dacron tube costs approximately 35,000 rupees (420.58 US dollars). Some institutions resort to pulmonary homografts, particularly those with access to homograft banks. These are available for free in institutions where these banks are available. However, availability is a challenge. In redo surgeries due to pericardial adhesions, harvesting a pericardial patch is of suboptimal quality, and bovine pericardial patches can be a better alternative for these patients. With the advancement of technology, promising bovine patches are available on the market. These patches can be used for various indications in the realm of cardiac redo surgeries and are available in various sizes.

Challenges in India regarding the provisions for redo pediatric cardiac surgery

Limited Understanding of Symptoms and the Need for Specialist Care

Healthcare professionals on the front lines and primary caregivers lack awareness about the challenges associated with CHD. Some of them hold the misconception that a child diagnosed with CHD is destined for a bleak future and may not experience a fulfilling life, even with appropriate interventions. The parents of these children who have undergone first-stage surgery become disillusioned and are lost to follow-up, resulting in greater morbidity and emergency redo surgery in certain situations [4].

Unequal Distribution of Resources

The treatment resources available for CHD not only fall short but also suffer from a significant imbalance in distribution. The geographical allocation of centers specializing in CHD surgeries is highly uneven. Moreover, the prevalence of poverty, a major impediment to the successful treatment of CHD, is notably higher in regions lacking adequate cardiac care facilities. In India, a substantial oversight pertains to the transportation challenges faced by newborns and infants with CHD [5]. Families dealing with a child who has CHD and needs surgery face a two-fold financial challenge. This includes not only the direct expenses incurred at the hospital but also the indirect costs related to provisions like food, transportation, accommodation, and salary loss experienced by caregivers during their hospital stay. Regrettably, the latter aspect, although crucial, often goes unnoticed by the medical treatment team [6].

Financial Constraints

In India, medical insurance coverage for birth defects is extremely limited, leading to a considerable financial burden for families, particularly in cases requiring redo surgeries. While various state-level government programs, microfinance initiatives, and charitable organizations aim to support economically disadvantaged sections of society, there is a pervasive lack of awareness within the community regarding these beneficial programs.

Lack of Follow-Up Care

Long-term care is essential for children with CHD, even after interventions to ensure favorable outcomes. Inadequate follow-up for children, particularly those from lower socioeconomic backgrounds, is a critical concern in India. With the healthcare system lacking proactiveness, the responsibility for follow-up rests solely on families, potentially jeopardizing the health and well-being of these children.

Additional Considerations

**Figure 1:**
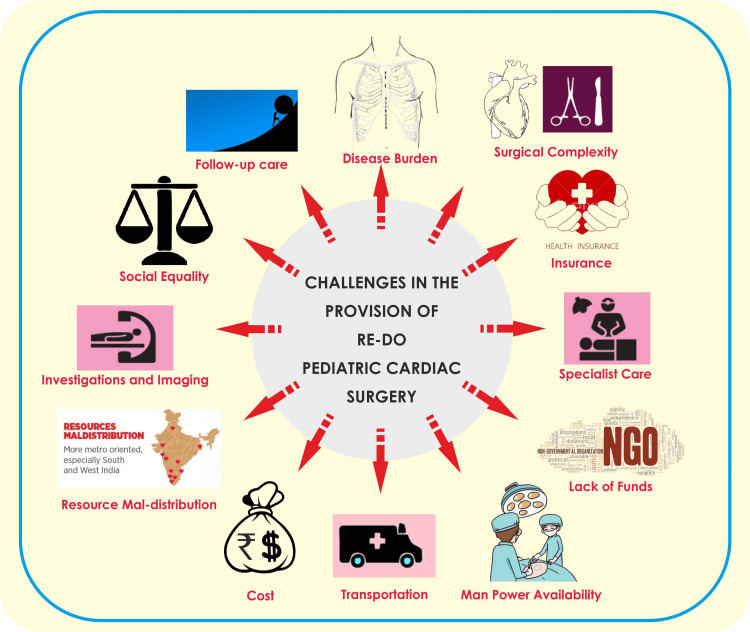
Challenges in the provisioning for redo surgery Image created by author Bhende

Numerous nonmodifiable factors contribute to the expenses associated with congenital heart surgery. Preoperative health status and surgical complexity were identified as the main determinants of outcomes in these pediatric cases. Study findings indicate that the total expenses rise in correlation with the complexity of the surgeries (Figure 1) [7,8].

Conclusions

Repeating a surgical procedure typically involves increased risks during the operation. However, conducting a thorough preoperative evaluation of the patient, employing suitable diagnostic instruments to delineate operative strategies, and exercising caution during mediastinal dissection offers optimal prospects for a positive outcome in redo cardiac surgery. A growing number of repeat interventions within this field are being addressed in pediatric cardiology, thereby simplifying the tasks for pediatric cardiac surgeons.
